# Relationship between Physical Exercise Self-Efficacy and Persistent Exercise Behavior among College Students

**DOI:** 10.31083/AP38955

**Published:** 2025-03-12

**Authors:** Ziao Hu, Yu Zhang, Chen Liao, Liying Nong, Kudulike Kadier, Kun Zhu

**Affiliations:** ^1^Department of Ophthalmology, The Second Xiangya Hospital of Central South University, 410011 Changsha, Hunan, China; ^2^Zhangjiajie College, Jishou University, 427000 Zhangjiajie, Hunan, China; ^3^Normal College, Hezhou University, 542899 HeZhou, Guangxi, China; ^4^Business School, Jishou University, 416000 Jishou, Hunan, China; ^5^Graduate School, St. Paul University, 3500 Tuguegarao, Philippines

**Keywords:** college students, physical exercise self-efficacy, cognitive, emotional, and behavioral engagement, persistent exercise behavior

## Abstract

**Background:**

Understanding the factors that sustain physical activity among college students is crucial for promoting a healthy lifestyle, as emphasized by the United Nations’ Sustainable Development Goal 3. While the link between physical activity and health outcomes is established, less is known about how physical exercise self-efficacy (PESE) influences persistent exercise behavior (PEB) through different forms of physical activity engagement (PAE). This study investigates whether PESE promotes PEB via cognitive, emotional, and behavioral engagement, based on self-determination theory (SDT) and engagement theory.

**Methods:**

An online cross-sectional survey was conducted from July 16 to August 16, 2023, involving 900 Chinese college students recruited through convenience sampling on the Questionnaire Star platform, the participants were selected through convenience sampling, which ensured the inclusion of a diverse demography across various grades, educational backgrounds, and study fields. Before the participants provided informed consent, they were briefed on the study’s objectives, data processing procedures, and privacy protections. Validated scales assessed PESE, PAE, and PEB. Data quality was ensured by excluding invalid or incomplete responses. Statistical analyses were performed in several stages. Using SPSS, item and reliability analyses of the research instrument were performed to confirm internal consistency. Then, the confirmatory factor analysis was performed for each scale by using AMOS. Finally, structural equation modeling was used to validate the proposed research model and conduct path analysis, thereby assessing the hypothesized relationships among PESE, PAE, and PEB.

**Results:**

PESE positively influenced cognitive, emotional, and behavioral engagement in physical activity. These forms of engagement, in turn, positively affected fluency experience (FE), which subsequently enhanced PEB. The findings indicate that PESE augments FE by improving PAE, leading to sustained exercise behavior among college students.

**Conclusions:**

The study demonstrates the critical role of PESE in fostering persistent exercise behavior through its impact on cognitive, emotional, and behavioral engagement. These insights highlight the importance of designing interventions that enhance PESE and PAE to promote long-term commitment to physical activity among college students, supporting broader health and well-being goals.

## Main Points

∙ Significance of physical exercise self-efficacy: Physical exercise self-efficacy is a critical motivational factor influencing the participation of college students in physical activities, which enhances their cognitive, emotional, and behavioral engagement, thereby contributing to the achievement of Sustainable Development Goals.

∙ Empirical Findings: The study validates seven research hypotheses and constructs a research model demonstrating that higher physical exercise self-efficacy among students increases engagement and enjoyable physical activity experiences, which, in turn, promote sustained exercise behavior.

∙ Impact of Engagement: Cognitive, emotional, and behavioral engagement in physical activities enhances the experience and significantly contributes to sustained physical exercise behaviors. This suggests that these engagements are critical for maintaining physical behaviors.

## 1. Introduction

The Sustainable Development Goals (SDG-3), focusing on promoting healthy lifestyles and individual well-being, have led to the prioritization of health and well-being since the introduction of SDG-3 in 2015 by the United Nations (UN) [1]. The worldwide outbreak of COVID-19 in 2019 has profoundly affected global health and SDG-3 implementation [2]. According to the World Health Organization, the lack of physical activity increases the risk of physical and mental health issues [3]. These risks were exacerbated during the COVID-19 pandemic period, affecting the implementation of SDGs [4]. In the post-pandemic global landscape since 2021, restrictions on physical activity among Chinese university students have been relaxed owing to the decrease in infection risks and relaxation of campus prevention and control measures. Nonetheless, disruptions to the established physical exercise routines of students during the pandemic have had a notable impact on their physical and mental health [5]. Epidemic infections can contribute to health issues such as physical deficiencies, potentially promoting unhealthy lifestyle habits that increase the risk of further epidemics and adversely affect individuals’ physical and mental well-being [6].

Engaging in physical activity is an effective means for improving overall health. Specifically, adequately engaging in exercises such as aerobic training and activities aimed at managing or reducing body weight can help mitigate the adverse effects of a sedentary lifestyle, an inevitable outcome of the pandemic, on mental and physical health [7]. Although the COVID-19 pandemic has led to decreased physical activity among students, those engaging regularly in exercises are likely to be benefited in terms of their physical and mental health [4]. This implies that greater persistent exercise behavior (PEB) is essential for physical health of college students and a continued active status. Despite the numerous obstacles encountered in the implementation of SDG-3, addressing how to improve PEB among college students to help them sustain a healthy lifestyle has emerged as a critical factor for achieving SDG-3.

Recently, greater emphasis has been placed on physical activity engagement (PAE) and health promotion [8, 9]. Motivation is a key driver of physical activity. It not only enhances PAE but also exerts a continuous and dynamic impact on physical activity-associated behavioral outcomes [10]. Furthermore, according to the self-determination theory (SDT) [11], people’s motivation is closely linked to their behavioral outcomes. Intrinsic motivation tends to influence individuals’ behavioral outcomes continuously through their intrinsic satisfaction. Self-efficacy is an individual’s confidence in their capacity to achieve specific objectives [12]. Within the sports psychology domain, physical exercise self-efficacy (PESE) reflects an individual’s confidence and beliefs in their ability to constantly engage in physical activities or exercises over time [13]. PESE is regarded as an essential motivator for individuals to engage in physical activity [14]. Higher levels of PESE are closely linked to increased physical activity. Generally, individuals with higher PESE levels are more confident in their ability to engage in physical activities, which possibly encourages them to invest more time and effort into such activities [15]. Elevated levels of PESE enhance students’ engagement in physical activity and enrich their participation experience [16]. This constantly influences their PEB. Consequently, this research explored the relationship between PESE and PEB among college students.

Furthermore, the engagement theory proposed by Fredricks *et al*. [17] describes individual engagement as a multidimensional construct encompassing cognitive, emotional, and behavioral dimensions. Engagement plays a crucial role in the motivational process and is closely linked with individuals’ environments and activities, affecting their behavior and outcomes in a consistent and dynamic manner [2, 17]. A study observed that the individuals displaying cognitive, emotional, and behavioral engagement (CC, EE, and BE, respectively) in physical activities exhibited improved behavior and health outcomes [2]. Given the lack of sufficient physical activity and exercise behavior among college students, PESE may serve as a crucial motivator for increasing their PAE [18]. Elevated PESE boosts the confidence of college students to involve in physical activities, potentially increasing their activity levels. It can also shift the perceptions of students toward prioritizing physical fitness and health promotion [15]. Therefore, examining the connections between PESE, PAE, and PEB among college students is critical.

Based on the preceding analysis, boosting college students’ PESE would motivate them for PAE and improve their FE during these activities. These improvements, in turn, would be conducive to enhanced PEB in students for sustaining a healthy lifestyle. PESE has been identified as a key motivator for PAE and PEB among college students. Engagement theory conceptualizes engagement as a multidimensional construct that encompasses cognitive, emotional, and behavioral dimensions. Engagement is pivotal for motivation, as it dynamically interacts with individuals’ environments and activities, thus influencing their behaviors and outcomes. Despite extensive reports highlighting the benefits of physical activity, a notable gap exists in the literature focusing on specific pathways through which PESE affects PAE and PEB among college students. Elevated PESE not only enhances students’ confidence in their ability to participate in physical activities but also modifies their perceptions toward prioritizing physical fitness and health status. However, the mechanisms through which PESE transforms into sustained exercise behavior through different PAE types remain underexplored [19, 20]. The present study, grounded in the principles of the SDT and engagement theory [17], examined the relationship between PESE, three PAE types, FE, and PEB among college students.

According to the SDT, individuals’ intrinsic and extrinsic motivations are intricately linked to their behavioral outcomes and constantly influence their behavior by stimulating the feelings of satisfaction and enhancing emotional experience [11]. Additionally, PESE, a major motivational factor, may influence students’ behavior by increasing their PAE [21]. Therefore, this study considered PESE as the “motivation”, FE as the “satisfaction”, and PEB as the “behavior”, hypothesizing that the increase in the PESE levels of college students may enhance their FE in physical activity, thereby elevating their PAE and PEB.

**Fig. 1.**
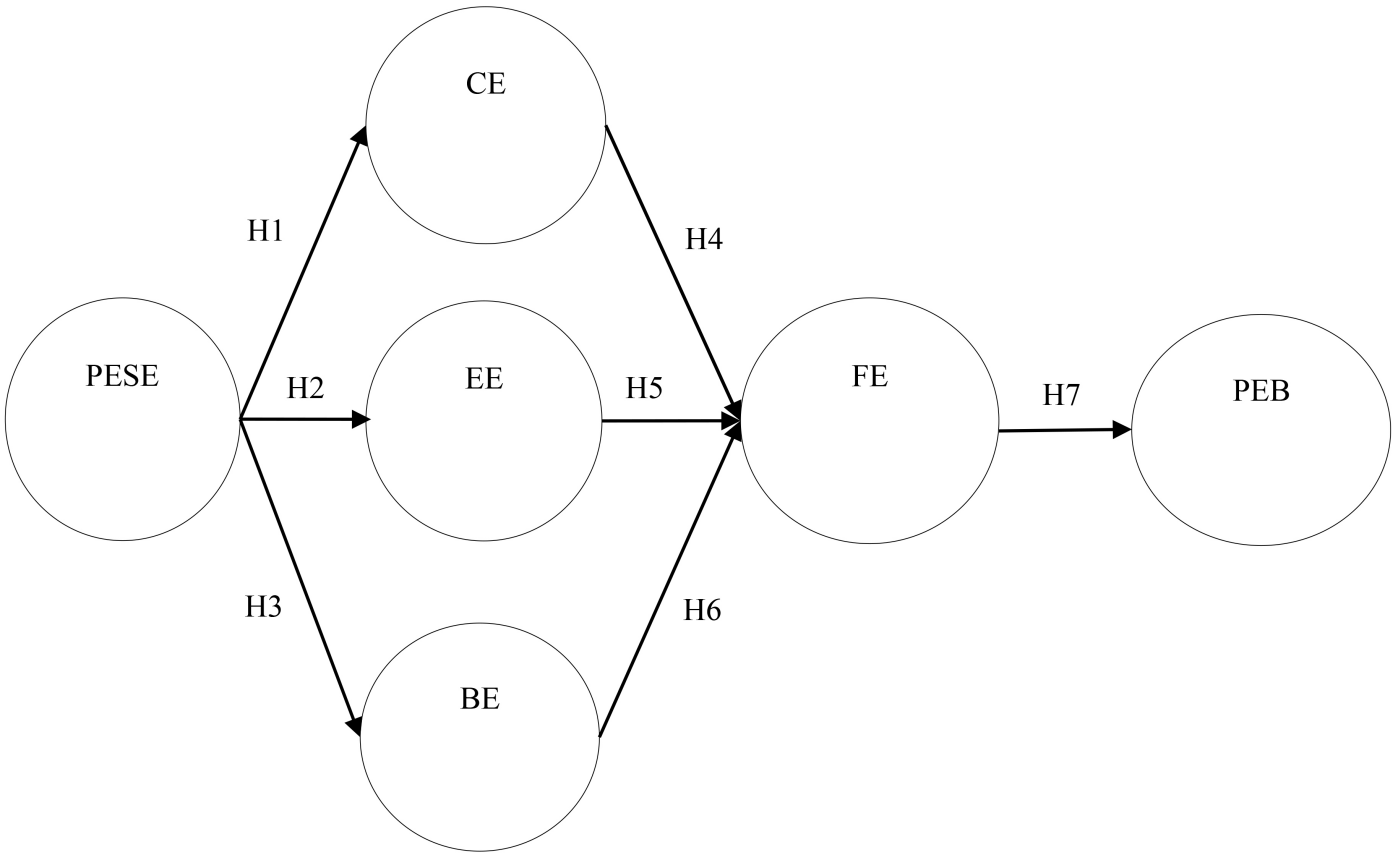
**Research Model**. Notes: PESE, physical exercise self-efficacy; CE, cognitive engagement; EE, emotional engagement; BE, behavioral engagement; FE, fluency experience; PEB, persistent exercise behavior.

According to engagement theory, the engagement is a construct encompassing CE, EE, and BE that closely, continuously, and dynamically influences people’s behavioral outcomes by motivating them to participate [17]. Consequently, greater CE, EE, and BE in physical activity may be more favorable for individuals’ participation motivation and consistently influence their PEB by enhancing their FE. To fully grasp the internal mechanism underlying the promotion of PEB in college students, this study assessed the relationship between PESE and three PAE types, FE, and PEB based on the SDT and engagement theory. A research model with seven hypotheses was developed (Fig. 1).

## 2. Research Hypotheses

### 2.1 Relationship between PESE and CE, EE, and BE in Physical Activity

SDT postulates that both intrinsic and extrinsic motivations influence individuals’ behavior and outcomes [11]. PESE reflects individuals’ confidence in their ability to engage in physical exercise, which significantly affects their motivation and behavior in physical activities [13, 14]. Studies have reported a strong association between PESE and PAE [16, 22]. CE refers to individuals’ knowledge and beliefs regarding physical activities, including their understanding of personal fitness [23]. EE involves emotional responses such as interest and excitement during physical activities [2], whereas BE relates to the frequency and manner in which individuals participate in physical activities [24]. PESE is crucial because it increases participants’ motivation for PAE and improves their fluency experience (FE), which, in turn, enables them to uphold a healthy lifestyle (PEB) through increased PAE [25, 26]. Despite the alleviation of the public health crisis, college students’ PAE remains low [8]. Enhancing their PESE could increase awareness, motivation, and initiative to participate in physical activities [27]. Therefore, the higher PESE of college students may not only improve their PAE but also influence their perceptions of the importance of participating in physical activity [25, 28]. Thus, such college students may participate in physical activity with greater CE, EE, and BE. Consequently, this study proposes the following hypotheses:

H1: PESE positively affects CE in physical activity.

H2: PESE positively affects EE in physical activity.

H3: PESE positively affects BE in physical activity.

### 2.2 Relationship of CE, EE, and BE with FE in Physical Activity

As we move toward normative management post-2024, promoting a healthy lifestyle is crucial for achieving SDG-3 [2, 29]. According to engagement theory [17], individuals’ CE, EE, and BE influence their motivation to participate in activities and their outcomes. Physical activity, as a key component of a healthy lifestyle, is associated with positive experiences and health benefits [30, 31]. Functional engagement (FE) in physical activity, which represents a comfortable and enjoyable experience, is linked to improved mood and health outcomes [32, 33].

Students regularly engaging in physical activity such as walking and running tend to have a higher motivation to participate [34, 35], which results in FE, excitement, and pleasure during physical activity [36]. In the current epidemic normalization management, college students have been recognizing the importance of physical activity [37]. Thus, they may feel compelled to participate in physical activities, which can enhance their likelihood of having enjoyable or seamless experiences during these activities [38]. Thus, the CE, EE, and BE of college students in physical activity tend to be associated with a greater FE. Accordingly, following hypotheses are proposed:

H4: CE positively affects FE in physical activity.

H5: EE positively affects FE in physical activity.

H6: BE positively affects FE in physical activity.

### 2.3 Relationship between FE in Physical Activity and PEB

With the increasing emphasis on achieving SDG-3, healthy lifestyles and the long-term health benefits of regular exercise have gained prominence [39]. PEB refers to consistent participation in activities like walking, running, and swimming, which is vital for promoting physical health among college students [40]. PEB is vital for college students because it significantly promotes their physical health [41]. Furthermore, college students regularly participating in physical activities with FE, such as excitement and pleasure, are more likely to engage in those activities [42]. Physically active college students tend to have higher participation experiences [43], which motivate them to engage constantly in physical activity and thus exhibit constant PEB [44]. Therefore, FE in physical activity is essential for such students as it allows them to uphold an exercise regimen. Thus, the following hypothesis is proposed:

H7: FE in physical activity is positively associated with PEB.

## 3. Methods

### 3.1 Research Procedure

In total, 900 Chinese college students participated in an online cross-sectional survey conducted using a questionnaire posted on Questionnaire Star platform from July 16, 2023, to August 16, 2023. The participants were selected through convenience sampling, which ensured the inclusion of a diverse demography across various grades, educational backgrounds, and study fields. Among the collected responses, only complete responses and those that fulfilled the inclusion criteria were considered valid for further analysis. Any legally consenting student enrolled in a Chinese university with internet access could participate in the survey. Before the survey was started, the participants were provided with detailed research-related information, including its purpose, potential benefits and risks, duration, and the procedures available for ensuring data confidentiality. The participants had to provide electronic informed consent for affirming their understanding and agreement to participate anonymously. This study was conducted according to the principles of the Declaration of Helsinki and its later amendments. The Ethical Committee of Zhangjiajie College, China, approved the study (ERB No. CLZCJSU-2023-01, Date: January 2023).

### 3.2 Participants

A total of 900 questionnaires were collected for this study. After excluding invalid responses, such as incomplete surveys or those completed in less than one minute, 845 valid questionnaires remained, resulting in an effective response rate of 93.9%. Respondents’ background information is detailed in Table 1.

**Table 1.** **Participants’ demographic backgrounds**.

Variable items	Content
Gender	536 females (63.4%).
	309 males (36.6%).
Grade	307 freshmen (36.3%).
	483 sophomores (57.2%).
	42 juniors (5%).
	13 seniors (1.5%).
Education	253 students in associate colleges (29.9%).
	243 students in universities (28.8%).
	201 students in vocational colleges (23.8%).
	148 students in vocational universities (17.5%).
Areas of Majors	10 students in Physical chemistry (1.1%).
	150 students in Arts and Humanities (17.7%).
	16 students in Social Sciences (1.9%).
	200 students in Computer and Engineering (23.7%).
	4 students in Nursing and Medicine (0.5%).
	36 students in Agriculture (4.3%).
	391 students in Business & Management (46.3%).
	38 students in Economics (4.5%).
Leisure Activities Usually Participate	83 students into outdoor static activities (9.9%).
	86 students into outdoor dynamics activities (10.2%).
	186 students into indoor static activities (22%).
	10 students into indoor dynamics activities (1.2%).
	95 students into socialization (11.2%).
	350 students into entertainment (41.4%).
	35 students into other activities (4.1%).
Days Per Week for Leisure Activities	193 students for none (22.9%).
	536 students for 1–2 days (63.4%).
	67 students for 3–4 days (7.9%).
	13 students for 5–6 days (1.5%).
	36 students for every day (4.3%).

## 4. Measure 

### 4.1 Physical Exercise Self-Efficacy (PESE)

PESE is an individual’s confidence and belief in completing the physical activity [13]. This study adapted Hong and Chan’s [45] self-efficacy scale based on the above definition to measure PESE among college students. The adapted scale in this study had 8 questions. Examples of questions were: “I believe that I can always complete the physical activity if I try hard enough” and “I am confident that I can effectively deal with any problems that arise during physical activity”.

### 4.2 Physical Activity Engagement (PAE)

PAE refers to the active cognitive, emotional, and behavioral involvement individuals exhibit when participating in physical activities [2, 46]. Drawing from the above definitions, this study designed 12 questions to assess physical activity engagement (PAE) among college students, based on Fredricks’ (2004) three types of engagement: cognitive (CE), emotional (EE), and behavioral (BE). Example questions for measuring CE in physical activity were: “I plan well in advance before performing physical activity” and “I remind myself to pay special attention to the areas where physical activity tend to be ineffective”. Example questions for measuring EE in physical activity were: “I am willing to improve my physical activity if it is not done in a good way” and “I like to discuss physical activity with my friends”. Example questions for BE in physical activity were: “I actively participate in physical activity” and “I am used to resting after participating in physical activity”.

### 4.3 Fluency Experience (FE)

FE in physical activity, found to be closely associated with positive emotions and health, refers to the feelings of excitement, comfort, and smoothness individuals experience during physical activity [32]. Based on the above definition, the study modified Jackson *et al*.’s [47] FE scale with 9 questions to measure FE in physical activity among college students. Example questions were: “I feel that everything goes well when I participate in physical activity” and “I am in optimal condition for physical activity”.

### 4.4 Persistent Exercise Behavior (PEB)

PEB is a behavioral manifestation in which an individual consistently exercises while performing physical activity [40]. Based on the above definition, 7 questions were developed to measure PEB among college students in this study. Example questions were: “I will continue to be physically active in the future” and “I will continue to pay attention to content about physical activity”.

### 4.5 Statistical Analysis

Structural equation modeling (SEM), a significant statistical method commonly used in social sciences, is often employed to examine the structure and hypothesized relationships among research variables [48]. Therefore, to investigate the relationships among the variables in the present study, SPSS 25.0 (IBM Corp., Chicago, IL, USA) was used for demographic background, correlation, and reliability analyses. Subsequently, AMOS 24.0 (IBM Corp., Armonk, NY, USA) was used to perform item analysis for each scale as well as to test model fit and conduct path analysis of the hypothesized research model by establishing the structural equation model.

## 5. Results and Discussion

### 5.1 Item Analysis

In social sciences studies, first-order confirmatory factor analysis (CFA) provides a better measure of item fitness and rationality of research scales [49]. According to the criteria recommended by statisticians, χ^2^/df should be less than 5, Goodness of Fit Index (GFI) greater than 0.80, Root Mean Square Error of Approximation (RMSEA) less than 0.1, and item questions with factor loading (FL) greater than 0.500 are statistically significant [50, 51]. Therefore, first-order confirmatory factor analysis (CFA) was conducted to assess the intrinsic validity of the study constructs, and the measured data met the criteria recommended by statisticians. Therefore, after the first-order CFA, measures for PESE were reduced from 8 to 7 questions; measures for CE in physical activity were reduced from 4 to 3 questions; measures for EE in physical activity were reduced from 4 to 3 questions; measures for BE in physical activity were reduced from 4 to 3 questions; measures for FE in physical activity were reduced from 9 to 6 questions; and no deleted questions for PEB measures, as shown in Table 2. The results of confirmatory factor analysis validated the measurement model.

**Table 2.** **First-order CFA**.

Constructs	χ^2^	*df*	χ^2^/df	RMSEA	GFI	AGFI	FL
Threshold value	-	-	<5	<0.10	>0.80	>0.80	>0.5
PESE	58.8	14	4.20	0.62	0.98	0.96	0.58–0.79
PAE	127.6	27	4.73	0.66	0.96	0.94	0.65–0.78
FE	27	9	3.00	0.49	0.99	0.98	0.74–0.85
PEB	43.92	14	3.14	0.92	0.99	0.97	0.60–0.79

Notes: RMSEA, Root Mean Square Error of Approximation; GFI, Goodness of Fit Index; AGFI, Adjusted Goodness of Fit Index; FL, Factor Loading; PESE, physical exercise self-efficacy; PAE, physical activity engagement; FE, fluency experience; PEB, persistent exercise behavior.

### 5.2 Reliability and Validity Analysis

In social statistical analysis, the reliability and validity of each research construct are assessed to evaluate the scientific rigor of the statistical data [50]. Regarding reliability, Cronbach’s α and composite reliability (CR) values greater than 0.7 indicate strong internal consistency within the constructs’ statistics. Table 3 demonstrates that the Cronbach’s α values for the constructs ranged from 0.92 to 0.94, and the composite reliability (CR) values ranged from 0.88 to 0.90, all exceeding 0.7. This indicates that the constructs possess excellent reliability and that the measured data meet the statistical standards [52]. Additionally, factor loading (FL) and average variance extracted (AVE) values greater than 0.5 indicate good convergent validity of the measured data [53]. In this study, FL values ranged from 0.71 to 0.78, and AVE values ranged from 0.50 to 0.61, both exceeding the 0.50 threshold. Furthermore, as shown in Table 4, the square root of the AVE for each construct is greater than its correlation coefficients with other constructs. This indicates good discriminant validity of the measured data [52]. Specifically, each construct shares more variance with its own measures than with other constructs, meeting the recommended criteria for discriminant validity [54]. The results showed that the constructs’ reliability and validity were appropriate.

**Table 3.** **Reliability and validity analysis**.

Constructs	M	SD	α	FL	CR	AVE	*t*
PESE	3.77	0.60	0.92	0.71	0.88	0.51	15.11–19.86
PAE	3.45	0.56	0.94	0.70	0.89	0.50	17.13–19.95
FE	3.49	0.61	0.94	0.78	0.89	0.61	21.91–25.71
PEB	3.70	0.59	0.92	0.74	0.90	0.56	17.55–24.61

Notes: M, Mean; SD, Standard Deviation; CR, Composite Reliability; AVE: Average Variance Extracted; PESE, physical exercise self-efficacy; PAE, physical activity engagement; FE, fluency experience; PEB, persistent exercise behavior.

**Table 4.** **Discrimination validity analysis**.

Constructs	1	2	3	4
(1) PESE	0.71			
(2) PAE	0.52	0.72		
(3) FE	0.54	0.73	0.78	
(4) PEB	0.59	0.65	0.77	0.75

Notes: PESE, physical exercise self-efficacy; PAE, physical activity engagement; FE, fluency experience; PEB, persistent exercise behavior.

### 5.3 Model Fitness Analysis

SEM typically assesses the variance acceptance of the resulting data by evaluating the overall fit of the study model [55]. Good overall fitness is indicated when the value of χ^2^/df is less than 5; the RMSEA is less than 0.1; the values of Goodness of Fit Index (GFI), Adjusted Goodness of Fit Index (AGFI), Normed Fit Index (NFI), Non-Normed Fit Index (NNFI), Comparative Fit Index (CFI), Incremental Fit Index (IFI), and Relative Fit Index (RFI) are greater than 0.800, and the values of Parsimony-Adjusted Normed Fit Index (PNFI) and Parsimony-Adjusted Goodness of Fit Index (PGFI) are greater than 0.500 [56, 57]. From the measured statistics, the following values were obtained for this study: χ^2^/df = 3.96, RMSEA = 0.06, GFI = 0.88, AGFI = 0.86, NFI = 0.89, NNFI = 0.91, CFI = 0.92, IFI = 0.92, RFI = 0.88, PNFI = 0.81, and PGFI = 0.75. These results indicated that the models in this study had good fitness and met the criteria recommended by statisticians.

### 5.4 Path Analysis

**Fig. 2.**
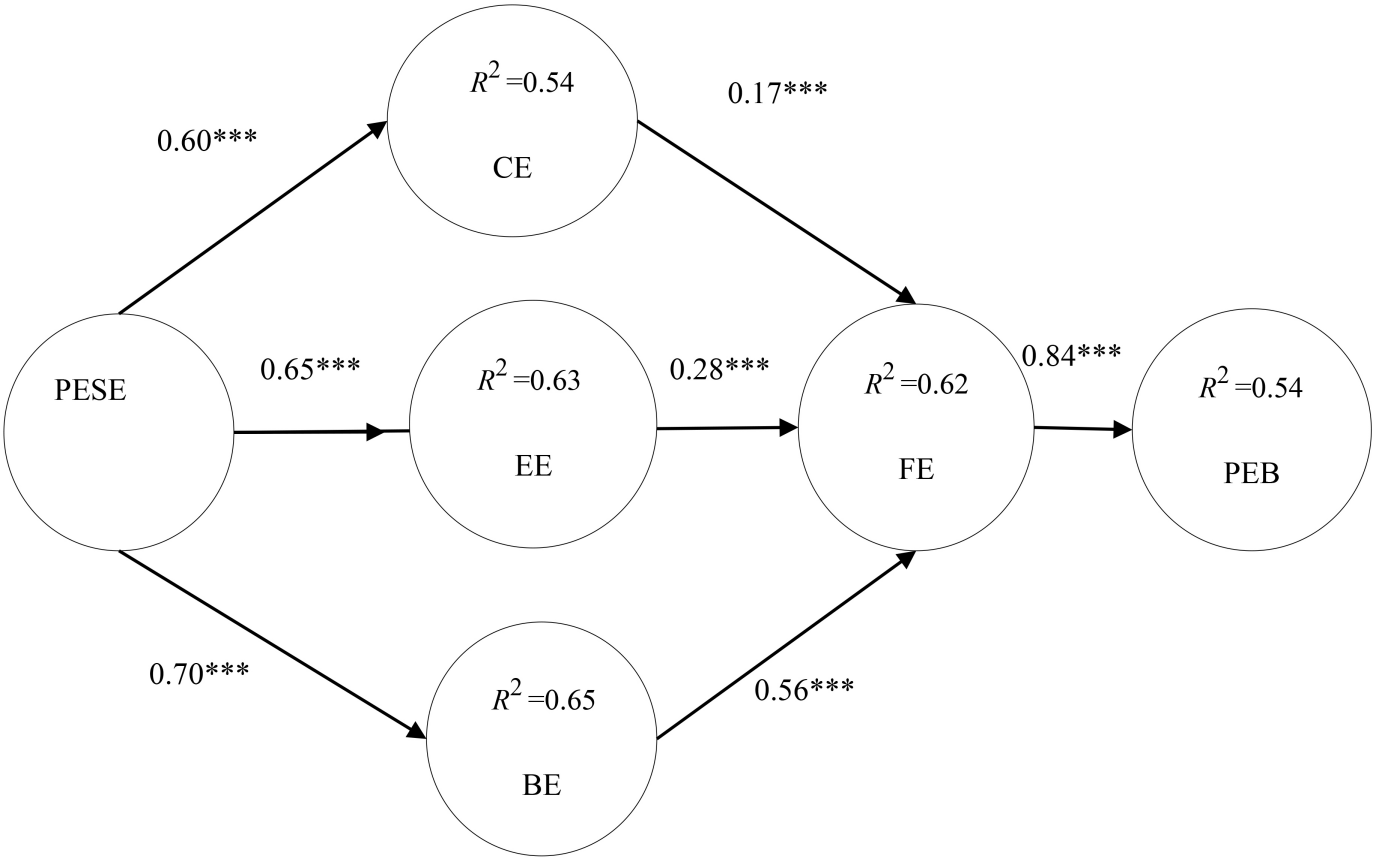
**Validation of the research model**. Notes: PESE, physical exercise self-efficacy; CE, cognitive engagement; EE, emotional engagement; BE, behavioral engagement; FE, fluency experience; PEB, persistent exercise behavior, *** *p* < 0.001.

This study proposed seven research hypotheses based on SDT and engagement theory to test the research model. As shown in Fig. 2, PESE had a significant positive effect on CE (β = 0.60***; t = 12.15), EE (β = 0.65***; t = 14.16), and BE (β = 0.70***; t = 14.43); CE (β = 0.17***; t = 4.73), EE (β = 0.28***; t = 7.73) and BE (β = 0.56***; t = 12.26) had a positive effect on FE; and FE had a positive effect on PEB (β = 0.84***; t = 19.73). In this context, the triple asterisks (***) indicate that the path coefficients are statistically significant at the *p* < 0.001 level.

In addition, the values of explanatory power ranging around 0.25, 0.50, and 0.75 indicate that the study model has weak, medium, and strong degrees of explanatory power, respectively [57]. As shown in Fig. 2, the explanatory power was 54% for CE, 63% for EE, 65% for BE, 62% for FE, and 54% for PEB. The aforementioned results indicated that the present study model had a moderate-to-strong degree of explanatory power.

## 6. Discussion

This study examined how PESE influences PEB among Chinese college students through cognitive, emotional, and behavioral engagement (PAE) in physical activity. Our findings provide new insights into the motivational processes that sustain exercise behavior, aligning with the SDT and engagement theory.

### 6.1 PESE Positively and Significantly Affects CE, EE, and BE in Physical Activity

The positive relationships between PESE and the three types of PAE underscore the crucial role of self-efficacy as a motivational factor in promoting physical activity among college students. This aligns with previous research findings indicating that higher PESE is closely related to increased participation in physical activities [22]. For example, Wang *et al*. [28] reported that college students with higher PESE are more likely to engage in physical activities and exhibit greater awareness. Similarly, Miller *et al*. [25] found that enhanced PESE improves emotional experiences, leading to increased enthusiasm for physical activity. The findings of the present study support the hypotheses 1, 2, and 3 by validating the significance of motivational factors of PESE in the three PAE types based on the SDT. In the context of promoting a healthy lifestyle and achieving SDG-3, our findings highlight the importance of fostering PESE among college students. Higher PESE motivates students to engage more in physical activity, contributing to their overall health and well-being. This is particularly significant during health crises, such as the COVID-19 pandemic, where maintaining physical activity is essential yet challenging.

### 6.2 CE, EE, and BE in Physical Activity Positively and Significantly Affect FE

Our study found that CE, EE, and BE in physical activity significantly and positively influence fluency experience (FE) among Chinese college students. This finding supports hypotheses 4, 5, and 6 and aligns with engagement theory [17], which posits that these forms of engagement are interconnected and dynamically influence behavioral outcomes. Additionally, these findings are consistent with those of previous research. Mitchell *et al*. [30] reported that increased engagement in physical activity enhances participation experiences, leading to greater involvement in activities like outdoor sports, swimming, and diving. Similarly, Nagata *et al*. [34] observed that students with higher levels of engagement in physical activities value their participation, which promotes excitement and pleasure, encouraging them to continue performing these activities. Regular engagement in physical activity enhances participation experiences, increasing motivation to maintain a healthy lifestyle. Based on the SDT, the positive relationship of CE, EE, and BE in physical activity with FE further supports the engagement theory [17], which posits that these forms of engagement are interrelated with the participation processes and pose a dynamic influence on behavioral outcomes.

### 6.3 FE in Physical Activity Positively and Significantly Affects PEB

To achieve the SDG-3, more attention needs to be paid to the influence of healthy lifestyles on health behavior [39]. Our results indicated that the FE in physical activity significantly and positively influences PEB among Chinese college students, supporting Hypothesis 7. This aligns with the findings of previous research indicating that positive participation experiences enhance individuals’ motivation to maintain routine physical activity [41]. For example, Ada *et al*. [42] reported that students with higher FE are more likely to engage consistently in activities like walking, running, and swimming. Similarly, Han *et al*. [44] discovered that college students with substantial participation experience remained self-motivated to engage in physical activity even during the COVID-19 pandemic. These findings highlight the importance of fostering positive experiences in physical activity to encourage sustained engagement. By enhancing FE, college students’ PEB can be promoted, contributing to healthier lifestyles and achieving SDG-3 focused on good health and well-being. Applying SDT, the present study enriches the understanding of how motivational factors like FE influence the maintenance of individual behaviors over time. The continued influence of FE on sustaining PEB underscores the need for interventions that enhance participation experiences to support long-term commitment to physical activity.

## 7. Conclusions

Grounded in the SDT and engagement theory, the present study investigated the interrelationships among PESE, the three types of PAE—CE, EE, and BE—FE, and PEB among Chinese college students. Our findings validated all seven research hypotheses, demonstrating that PESE is a vital motivational factor that enhances CE, EE, and BE in physical activity. This increased engagement leads to a more positive FE, which, in turn, promote sustained exercise behavior [5]. The results highlight that higher PESE is associated with greater cognitive, emotional, and behavioral investments in physical activity among college students, enhancing their FEs and reinforcing their motivation to maintain a consistent exercise routine. By elucidating the internal mechanisms through which PESE influences PEB via PAE and FE, the study offers deeper insights into how to promote healthy lifestyles in this population, aligning with the objectives of SDG-3 [58].

By elucidating the internal mechanisms through which PESE influences PEB via PAE and FE, this study provides a theoretical framework for understanding how motivational factors drive sustained physical activity. Furthermore, the use of SDT and engagement theory in this study to explain the linkages among PESE, PAE, FE, and PEB contributes to the existing literature by highlighting the role of self-efficacy in promoting health behaviors. In addition, this study provides empirical evidence for the role of PESE as a critical motivational factor that positively influences multiple dimensions of PAE. Specifically, higher PESE is linked to greater cognitive, emotional, and behavioral investment in physical activity among college students, ultimately enhancing their FEs. This insight underscores the importance of self-efficacy in motivating students to engage actively in physical activities, thus helping address the widespread issue of physical inactivity in this demographic. Given the persistent issues of sedentary lifestyles and physical inactivity among college students, our findings underscore the importance of fostering PESE and all forms of engagement to enhance FEs and support sustained physical activity. Educators and policymakers should consider implementing strategies that boost students’ confidence in their exercise abilities and encourage deeper engagement in physical activity.

Individuals engaging in physical activity can achieve a greater sense of comfort, which would enable them to sustain their physical activity behavior. Therefore, educators and college students should recognize the prominence of physical activity, dedicate more effort and enthusiasm to it, and value FE. This will further motivate them to participate in and sustain their physical activity. According to the SDT, intrinsic and extrinsic motivations and their correlations impact behavioral outcomes [11]. Extrinsic motivations, such as rewards and health, also influence people’s willingness to participate in physical activity, thereby impacting their experience [10, 59]. While the present study provides valuable insights, it has limitations. Its cross-sectional design restricts the ability to infer causality among the variables. Future research could employ longitudinal designs to explore causal relationships and examine the impact of both intrinsic and extrinsic motivational factors on PEB. Additionally, investigating the effects of PAE on negative behavioral outcomes could further enrich the understanding of engagement in physical activity [60]. In conclusion, this study confirms the roles of PESE, PAE, and FE in promoting PEB, providing valuable insights into the motivational processes that sustain physical activity among college students. These insights can inform the development of targeted interventions aimed at improving the health and well-being of young adults, echoing the objectives outlined in the introduction.

## Availability of Data and Materials

The data that support the findings of this study are available from the corresponding author.
